# Relationships among breast, gut, and oral microbiota across diverse pathological types of breast cancer, a Chinese cohort study

**DOI:** 10.3389/fmolb.2023.1325552

**Published:** 2023-11-21

**Authors:** Kexin Feng, Fei Ren, Xiang Wang

**Affiliations:** Department of Breast Surgical Oncology, National Cancer Center/National Clinical Research Center for Cancer/Cancer Hospital, Chinese Academy of Medical Sciences and Peking Union Medical College, Beijing, China

**Keywords:** breast cancer, fecal microbiome, non-malignant breast diseases, oral microbiome, Chinese

## Abstract

**Background:** Recent research has unveiled the association between microbiota and the onset and progression of breast cancer (BC). This study investigates the microbiota in breast tissue, the gut, and the oral cavity in relation to different pathological types of breast diseases, aiming to unveil the microbiota-BC relationship and provide new perspectives for BC diagnosis and treatment.

**Methods:** The study encompassed a total of 98 breast cancer patients, with 52 diagnosed with Luminal A BC, 17 with Luminal B BC, 18 with HER2 BC, and 11 with TNBC. In addition, there were 46 patients with non-malignant breast diseases. The V3-V5 region of the 16S rRNA gene of breast tissue, feces, and the oral cavity was sequenced. Based on Amplicon Sequence Variants (ASV) representative sequences and abundance information, a series of statistical analyses were conducted including community diversity analysis, community composition analysis, species difference analysis, correlation analysis, and functional prediction analysis.

**Results:** Notable divergences in α-diversity and β-diversity were discerned in breast tissue between BC patients and non-malignant breast disease patients. The linear discriminant analysis effect size (LEfSe) and random forest examinations pinpoint Pasteurellaceae as a significant predictor in BC cohorts. Further exploration revealed significant microbial distribution divergences across distinct pathological types of BC, with notable variations in the relative abundance of microbial species such as Streptococcus, Serratia, and Pseudomonas, underscoring the diverse microbial diversity across BC subtypes and sample origins.

**Conclusion:** This venture sheds light on the complex microbiota milieu across varying body sites and pathological types of BC, emphasizing microbiota-BC connectivity. This articulation of a multisite microbiota-BC interrelation significantly advances a holistic grasp of BC pathogenesis.

## 1 Introduction

Breast cancer (BC) reigns as the most prevalent cancer among women globally, with estimations in 2023 projecting nearly 300,000 women will be diagnosed and around 43,170 women will succumb to this ailment (Cancer Statistics, 2023), accentuating the critical necessity for in-depth etiological investigation to foster enhanced early detection and treatment strategies. The etiological foundations of BC, embodying a complex orchestration of genetic and environmental determinants, continue to be enigmatically nuanced. Hence, the imperatives of enhancing early diagnosis and orchestrating efficacious treatment strategies have emerged as pressing concerns.

The human microbiome, a refined and dynamic aggregate of microbial entities, exhibits its ubiquity across a plethora of anatomical niches, spanning from the epidermis to the oral and vaginal cavities. Emerging scientific paradigms robustly accentuate the profound impact of host microbiota in modulating the tumor microenvironmental ambit. Dysbiosis within this microbial aggregate can precipitate chronic inflammatory states, reconfigure immune responses, and potentially undermine genome fidelity, thereby instigating DNA aberrations and dysregulating metabolic pathways. Such perturbations furnish a fertile substrate for BC pathogenesis and advancement (Rossi et al., 2020; Sepich-Poore et al., 2021).

The intestinal tract harbors the most substantial contingent of bacterial flora within the human body (Yang et al., 2023). Pertinent seminal inquiries have elucidated disturbances in fecal microbiota diversity in individuals afflicted with BC, with a pronounced emphasis on bacterial taxa such as Clostridiaceae, Faecalibacteriaceae, Ruminococcaceae, Dorea, and Lachnospiraceae (Terrisse et al., 2021; Tzeng et al., 2021; Papakonstantinou et al., 2022). Furthermore, with its intricate complexity, the oral microbiome has been conjectured to play a contributory, albeit partial, role in BC ontogenesis (Nearing et al., 2023. Research suggests a potential link between periodontal disease and an elevated risk of breast cancer (Freudenheim et al., 2016). A meta-analysis of 11 studies demonstrated a significant elevation in breast cancer risk associated with periodontal disease, with a relative risk of 1.22 (Chung et al., 2016). Common pathogenic factors like microorganisms and inflammation, shared between periodontal disease and breast cancer, may impact the onset and progression of breast cancer (Zhang et al., 2023). Existing literature suggests a possible association between oral microbiota and breast cancer. Particularly, studies reveal variations in oral microbial communities between breast cancer patients and healthy women (Thu et al., 2023). Additionally, research identifies a potential association between oral microbiota, particularly those related to menopause and menstrual status, and breast cancer risk. Recent scholarly ventures have delved into the micro-ecology of breast tissue. Sepich-Poore et al.’s (Sepich-Poore et al., 2021) pioneering exploration meticulously cataloged the tumor microbiota across a spectrum of malignancies, unveiling a particularly diverse bacterial milieu in BC. Augmenting this discourse, Fu et al. (Fu et al., 2022) unearthed the hitherto unexplored presence of “intracellular bacteria” within BC tissues, a phenomenon with profound ramifications for metastatic inclinations. This revelation embodies a seismic shift in our ontological comprehension of tumor metastasis. Moreover, the nuanced interplay between microbial niches, as epitomized by the presence of *Fusobacterium* nucleatum, an oral anaerobe with established oncogenic tendencies, in breast cancer, further accentuates the complexity of these interactions (Parhi et al., 2020; Little et al., 2023). More specific research indicates an interaction between the microbial communities of the gut and breast, potentially impacting breast health and breast cancer development. For instance, gut microbes may be transmitted to the breast, potentially altering the breast microbial community and thereby affecting the development of breast diseases (Zhang et al., 2021).

In the realm of composite research on microbiota across diverse body regions, presently, there exists solely one study investigating the oral and fecal microbiota within the Ghanaian populace (Wu et al., 2022), and another delving into the microbiota of breast tissue, oral cavity, and urine (Wang et al., 2017). All remaining studies focus on single-site microbial analyses. The two aforementioned studies exclusively target breast cancer patients and healthy control cohorts. As of now, no research has concurrently explored the oral, gut, and breast microbiota of breast cancer patients, nor ventured into examining microbiota variations across different body regions for diverse pathological types of breast cancer. Aiming to bridge this knowledge gap, our study leverages the precision of 16S sequencing to evaluate fecal, saliva, and breast tissue specimens across a broad spectrum of BC pathologies. This comprehensive analysis aims to elucidate the complex bacterial interactions within these specific anatomical regions and explore their potential implications for breast cancer pathogenesis. Consequently, the notion of an oral-gut-breast axis has been proposed, wherein dysregulated oral bacteria infiltrate the gut, trigger adverse events in the resident breast microbiome, and contribute to breast diseases. The insights gleaned from this inquiry hold the potential to fundamentally reshape existing paradigms, thereby propelling the development of tailored therapeutic approaches in the realm of breast cancer.

## 2 Materials and methods

### 2.1 Patient enrollment and tissue collection

The investigation was carried out at the National Cancer Center/National Clinical Research Center for Cancer/Cancer Hospital, Chinese Academy of Medical Sciences, and Peking Union Medical College during the timeframe from January 2022 to March 2022. The confirmation of breast cancer diagnoses was achieved through ultrasonography, radiography, or breast MRI, and further substantiated by fine needle aspiration or core needle biopsy of breast tissue. Individuals were omitted from the study if they were below 18 years old, had a history of other malignancies, suffered from oral afflictions, had been treated with antibiotics or probiotics within the preceding 2 months, or lacked complete data. The exclusion criteria for control subjects mirrored those for patients, with additional exclusions for gastrointestinal maladies, history of malignancies, chronic non-communicable diseases, or incomplete data.

### 2.2 Sample collection and storage

Salivary specimens were obtained utilizing the Salivettes^®^ sampling apparatus (Sarstedt, Nümbrecht, Germany) promptly upon participants’ arousal (between 7 and 8 a.m.), with 5–10 mL being collected. Fecal samples were self-collected pre-operatively using stool sampling kits. Fresh-frozen breast tissues were acquired in adherence to standard biorepository protocols from individuals undergoing surgical intervention for breast cancer. A specimen container, housing 5 mL of sterile saline or water, was unveiled in the surgical suite during breast surgery to mitigate potential microbial contamination from the environment. Concurrently with tissue specimens, these environmental controls were processed. All specimens were preserved at −80°C within a 4-h window post-receipt, pending conveyance to the laboratory for processing and analysis.

### 2.3 16S rRNA gene sequencing

#### 2.3.1 Extraction of genomic DNA

Genomic DNA was extracted from the specimens employing the CTAB/SDS methodology. The concentration and purity of DNA were ascertained by electrophoresis on a 1% agarose gel. DNA was diluted to a concentration of 1 ng/μL using sterile water, the specific dilution factor being contingent on the initial concentration.

#### 2.3.2 Amplicon generation

Ultrapure water was utilized in the DNA extraction procedure to preclude false-positive PCR outcomes. The 16S rRNA genes from varied loci, namely, 16S V4, 16S V3, 16S V3-V4, and 16S V4-V5, were amplified employing designated primers (e.g., 16S V4: 515F-806R et al.) inclusive of barcodes. The PCR reactions comprised 15 µL of Phusion^®^ High-Fidelity PCR Master Mix, 0.2 µM of both forward and reverse primers, and approximately 10 ng of template DNA. The thermal cycling regimen entailed an inaugural denaturation step at 95°C for 4 min, succeeded by 30 cycles, each encompassing denaturation at 95°C for 10 s, annealing at 50°C for 30 s, and elongation at 72°C for 30 s, culminating with a 72°C hold for 4 min. Ultrapure water was utilized in the DNA extraction procedure to preclude false-positive PCR outcomes. The 16S rRNA genes from varied loci, namely, 16S V4, 16S V3, 16S V3-V4, and 16S V4-V5, were amplified employing designated primers (e.g., 16S V4: 515F-806R et al.) inclusive of barcodes. The PCR reactions comprised 15 µL of Phusion^®^ High-Fidelity PCR Master Mix, 0.2 µM of both forward and reverse primers, and approximately 10 ng of template DNA. The thermal cycling regimen entailed an inaugural denaturation step at 95°C for 4 min, succeeded by 30 cycles, each encompassing denaturation at 95°C for 10 s, annealing at 50°C for 30 s, and elongation at 72°C for 30 s, culminating with a 72°C hold for 4 min.

#### 2.3.3 PCR product quantification and qualification

An equal volume of 1× loading buffer was amalgamated with the PCR outputs, followed by electrophoresis on a 2% agarose gel for detection. PCR products were pooled in equimolar proportions, thereafter undergoing purification via the Qiagen Gel Extraction Kit.

#### 2.3.4 Library preparation and sequencing

Sequencing libraries were generated employing the TruSeq^®^ DNA PCR-Free Sample Preparation Kit, in congruence with the manufacturer’s stipulations. Index codes were integrated into the libraries. Library quality was evaluated using the Qubit@ 2.0 Fluorometer and the Agilent Bioanalyzer 2100 apparatus. The library was sequenced using Illumina NovaSeq technology, yielding 250 base pair paired-end reads. Initially, quality control and filtering, based on sequencing quality, are performed on the paired-end reads. Concurrently, the overlapping relationships between reads are utilized for assembly, yielding optimized data post-assembly. Subsequently, the DADA2 sequencing denoising method is utilized to obtain Amplicon Sequence Variant (ASV) representative sequences and abundance information. Utilizing the ASV representative sequences and abundance information, a series of statistical or visual analyses are conducted, including taxonomic, community diversity, community composition, species difference, correlation, and functional prediction analyses.

### 2.4 Clinicopathologic parameters

Baseline clinical and pathological data were documented, encompassing patients’ age, pre-existing basal diabetes, hypertension and diabetes history, body mass index (BMI), along with pathological and surgical details. TNM staging was evaluated according to the guidelines set forth in the 8th edition of the American Joint Committee on Cancer (Giuliano et al., 2018). Based on the results from immunohistochemistry tests, the statuses of estrogen receptor (ER) and progesterone receptor (PgR) were established. A positive hormone receptor (HR) status was identified if either ER or PgR tested positive in immunohistochemistry, while a negative status was recorded if both were negative. The classification of Human Epidermal Growth Factor Receptor 2 (HER2) was determined as negative when immunohistochemistry showed negative or 1+ results, and positive for 3+ results; for results of 2+, HER2 positivity was ascertained through fluorescence *in situ* hybridization findings. The calculation of Body Mass Index (BMI) was performed as the ratio of body weight (in kilograms) to the square of height (in meters). The surgical interventions in breast tissue were distinguished as either lumpectomy or mastectomy, contingent upon the extent of surgery.

## 3 Statistical analysis

Bioinformatics and statistical analysis were performed on the derived sequence data. The DADA2 method (Callahan et al., 2016), contained within the QIIME2 software (Bolyen et al., 2019), was employed for denoising and the acquisition of ASVs. Normalized ASV abundance tables were utilized as the foundation for subsequent analyses. Alpha diversity (α-diversity) primarily assesses community diversity within a specific ecological environment or sample, utilizing indices like Chao1, Shannon, and Simpson to gauge species richness and diversity. Beta diversity (β-diversity) analysis, conducted with QIIME2 software, evaluates the similarity in species diversity across different samples. Beta diversity analysis primarily utilizes four algorithms: Binary Jaccard, Bray Curtis, Weighted Unifrac, and Unweighted Unifrac, to compute the distance between samples and derive β values. These four algorithms are divided into two main classes: weighted (Bray-Curtis and Weighted Unifrac) and unweighted (Jaccard and Unweighted Unifrac). Unweighted methods primarily compare the presence or absence of species; a smaller β diversity value between two communities indicates higher species similarity. Weighted methods consider both the presence or absence, and the abundance of species. Linear discriminant analysis Effect Size (LEfSe) identifies species features that best explain inter-group differences among two or more groups of samples under varying biological conditions or environments, and evaluates the extent of these features’ impact on the differences. PICRUSt2 estimates functional composition of microbial communities using marker genes. KEGG is a database integrating genomic, chemical, and systemic functional information for interpreting biological systems. Following this, random forest analysis was conducted, and receiver operating characteristic (ROC) analysis was performed using the pROC package based on the results of the random forest analysis. All statistical analyses were performed using the R program (Version 4.2.2), with a *p*-value of <0.05 denoting statistical significance.

## 4 Results

### 4.1 Baseline characteristics of the participants

**FIGURE 1:**
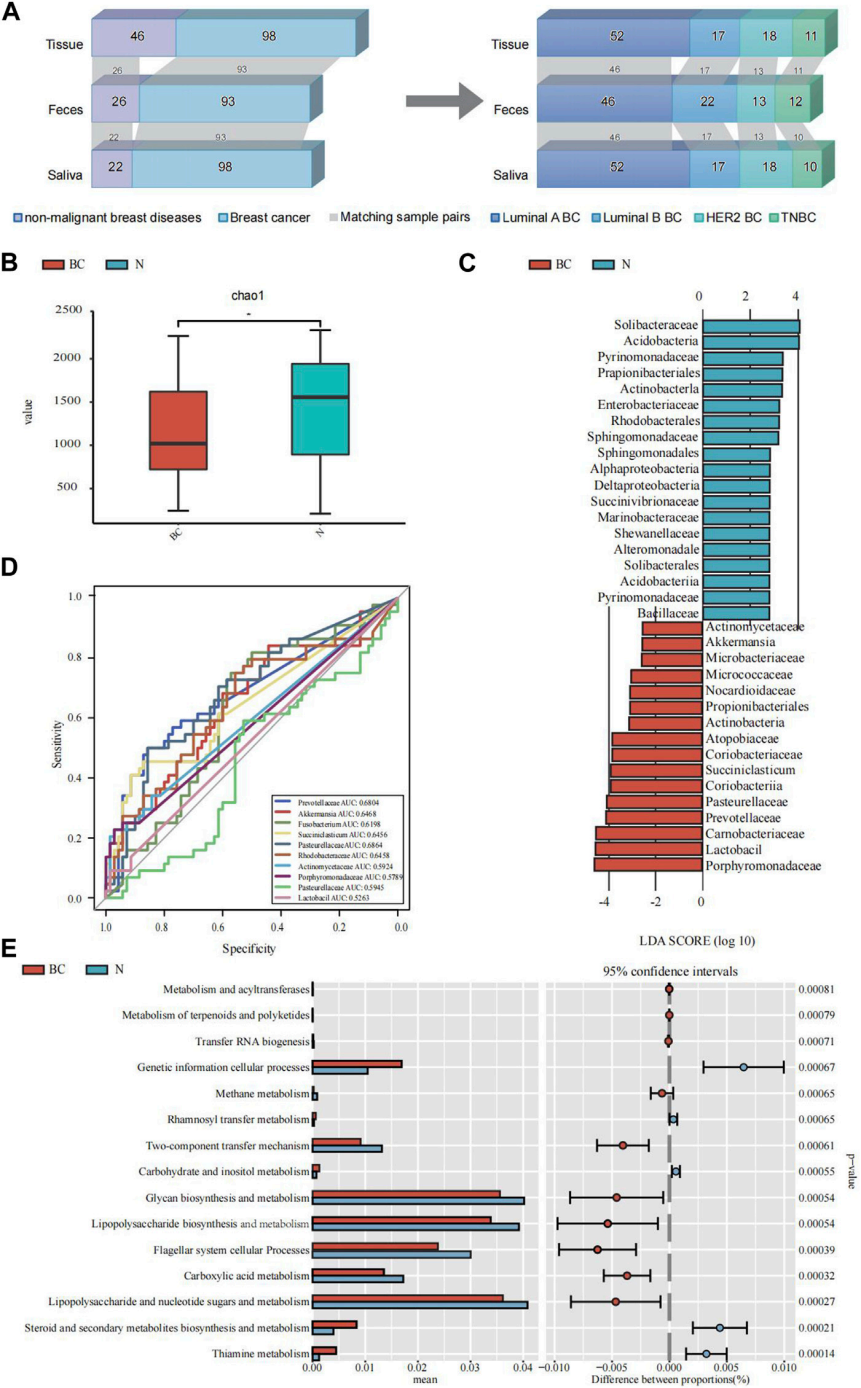
An illustrative delineation of the study cohort and the microbial composition and difference between breast cancer (BC) and the non-malignant groups (N). **(A)** Overview of the study population. Grey bands between bar plots represent samples of matching body regions within individuals. **(B)** Alpha diversity index (Chao1 indices) of for the breast tissue samples. **(C)** The common characteristic bacteria of the BC and N groups found in LEfSe of breast tissue samples. **(D)** The top 10 genera were tested by ROC analysis of BC in breast tissue samples. **(E)** The major KEGG pathways between BC and the N groups with the 16S sequencing data of breast tissue samples. Differential shotgun metagenomic sequence-based KEGG pathways in microbiota between the two groups detected by Diamond software. The top 15 items are listed along with the appropriate 95% confidence intervals and adjusted-p values.

The study encompassed a total of 98 breast cancer patients, with 52 diagnosed with Luminal A BC, 17 with Luminal B BC, 18 with HER2 BC, and 11 with TNBC. In addition, there were 46 patients with non-malignant breast diseases. The baseline characteristics of the cohort are in Table 1. For some of these patients, fecal and saliva samples were paired. An illustrative delineation of the study cohort is presented in Figure 1A.

**TABLE 1:** The clinical characteristics of patients with breast cancer and non-malignant breast diseases.

Characteristics	Breast cancer (N = 98)	Non-malignant breast diseases (N = 46)	*p*-Value
Age, years Median (IQR)	49 (18.5)	54 (15.25)	0.163
BMI—kg/m^2^ Median (IQR)	24.46 (5.01)	24.8 (5.47)	0.460
Smoking status			0.109
Never smoker	95 (96.9%)	40 (87.0%)	
Former smoker	3 (3.1%)	6 (13.0%)	
Current smoker	0	0	
Alcohol consumption			0.711
Never drink	65 (66.3%)	29 (63.0%)	
	33 (33.7%)	17 (37.0%)	
1 standard drink per day	0	0	
Diabetes			0.212
Yes	48 (49.0%)	28 (60.9%)	
No	50 (51.0%)	18 (39.1%)	
Oral contraceptives use past			0.854
Yes	36 (36.7%)	18 (39.1%)	
No	62 (63.3%)	28 (60.9%)	
Number of live births			0.475
0	33 (33.7%)	12 (26.1%)	
1–2	49 (50%)	28 (60.9%)	
≥3	16 (16.3%)	6 (12.5%)	

### 4.2 Diversity of microbial abundance among breast cancer and healthy controls

Initially, comparative scrutiny of microbial compositions across three sample types from individuals with BC and those with non-malignant breast ailments was performed.

Employing α-diversity and β-diversity as pivotal descriptors, we gained a more nuanced understanding of the microbiota’s overall assembly and dispersion in relation to BC susceptibility. Table 2 illustrates the comparison of microbial diversity indices in breast tissue, fecal, and saliva samples between breast cancer patients and patients with non-malignant breast diseases. In breast tissue samples, we observed significant differences in α-diversity indices between breast cancer patients and non-malignant cases for the following metrics: Chao1 (*p* = 0.019, Figure 1B), ACE (*p* = 0.019), Shannon (*p* = 0.015), and Simpson (*p* = 0.021). The indicators of α-diversity of microbial communities in breast tissue, fecal and saliva samples are in Supplementary Figure S1. Additionally, β-diversity indices, namely, Unweighted UniFrac (*p* = 0.032) and Weighted UniFrac (*p* = 0.019), also demonstrated significant disparities between breast cancer patients and non-malignant cases. In feces and saliva samples, no significant differences were observed in the breast tissue, feces, or saliva samples between breast cancer patients and patients with non-malignant breast diseases. The indicators of β-diversity of microbial communities in breast tissue, fecal and saliva samples are in Supplementary Figure S1. The heatmaps of species composition (at the genus level) of the three samples is in Supplementary Figure S2.

**TABLE 2:** Comparison of microbial diversity indices in tissue, feces, and saliva samples between breast cancer patients and patients with non-malignant breast diseases.

Microbial diversity index	Breast cancer cases VS. non-malignant cases		
	Tissue	Feces	Saliva
Alpha diversity index			
Chao1	0.019	0.490	0.630
ACE	0.019	0.440	0.650
Shannon	0.015	0.560	0.770
Simpson	0.021	0.630	0.920
Beta-diversity index			
Bray-Curtis	0.166	0.147	0.240
Jaccard	0.084	0.096	0.069
Unweighted UniFrac	0.032	0.055	0.153
Weighted UniFrac	0.019	0.092	0.225

Since only the α-diversity and β-diversity of breast tissue samples showed significant differences, we conducted further analysis on the breast tissue samples using LEfSe and Random Forest, along with functional prediction analysis.

We employed the LEfSe analysis method to compare the microbial composition across different groups. Through Linear Discriminant Analysis (LDA), we estimated the effect size of abundance differences for each species (LDA score >3, Bonferroni-adjusted *p* < 0.05). The results indicated that Pasteurellaceae, Prevotellaceae, Carnobacteriaceae, Lactobacil, Porphyromonadaceae, and Actinomycetaceae exhibited significant abundance differences in the breast cancer group. A subsequent random forest examination of the top 10 genera within the BC cohorts pinpointed Pasteurellaceae as having a prime predictive acumen with an area under the curve (AUC) of 68.64%, followed by Prevotellaceae (68.04%), Akkermansia (64.68%), Rhodobacteraceae (64.58%), Succiniclasticum (64.56%) and *Fusobacterium* (61.98%) (Figure 1D). The PICRUSt2 analysis for KEGG pathway envisaged a marked decline in multiple metabolic activities in the BC cohort vis-a-vis the non-malignant cohort, like the carbohydrate and inositol metabolism, lipopolysaccharide and nucleotide sugars and metabolism, and steroid and secondary metabolites biosynthesis and metabolism (Figure 1E).

### 4.3 Diversity of microbial abundance among four subtypes of breast cancer

**FIGURE 2:**
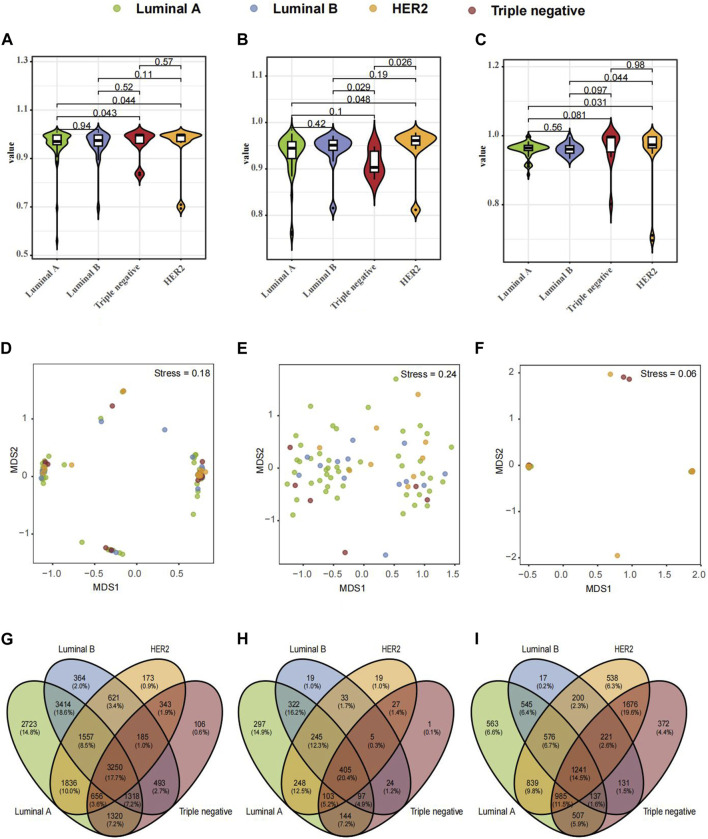
Composition and Differential Microbial Presence in Breast Tissue, Fecal, and Saliva Samples across Four Breast Cancer (BC) Types. **(A)** Simpson's α-diversity index for breast tissue samples. **(B)** Simpson's α-diversity index for fecal samples. **(C)** Simpson's α-diversity index for saliva samples. **(D)** NMDS-based β-diversity for breast tissue samples. **(E)** NMDS-based β-diversity for fecal samples. **(F)** NMDS-based β-diversity for saliva samples. **(G)** Venn diagram of microbial taxa in breast tissue. **(H)** Venn diagram of microbial taxa in fecal samples. **(I)** Venn diagram of microbial taxa in saliva samples.

Further exploration ensued on microbiological variances across three paired samples from BC-afflicted individuals showcasing diverse pathological types, including Luminal A, Luminal B, HER2, and TNBC. The Shannon diversity index of breast tissues, fecal and saliva samples, which considers both species richness and evenness, indicate a high level of diversity within four subtypes of breast cancer (Figures 2A–C). To visualize the dissimilarities in microbial community composition between samples, a non-metric multidimensional scaling (NMDS) analysis was performed based on Bray-Curtis dissimilarity. The analysis revealed a stress metric value of 0.24 and 0.06 for fecal and saliva microbiota and 0.18 for breast tumor microbiota, indicating the accuracy of the clustering results (Figures 2D–F). In analyzing microbial compositional differences across breast cancer subtypes, Venn Diagram identified unique microbial species in various samples (Figures 2G–I). Significant divergences in microbial distribution among distinct pathological types within all three samples were evident, elucidating the distinctive microbial diversity across BC subtypes and sample origins.

**FIGURE 3:**
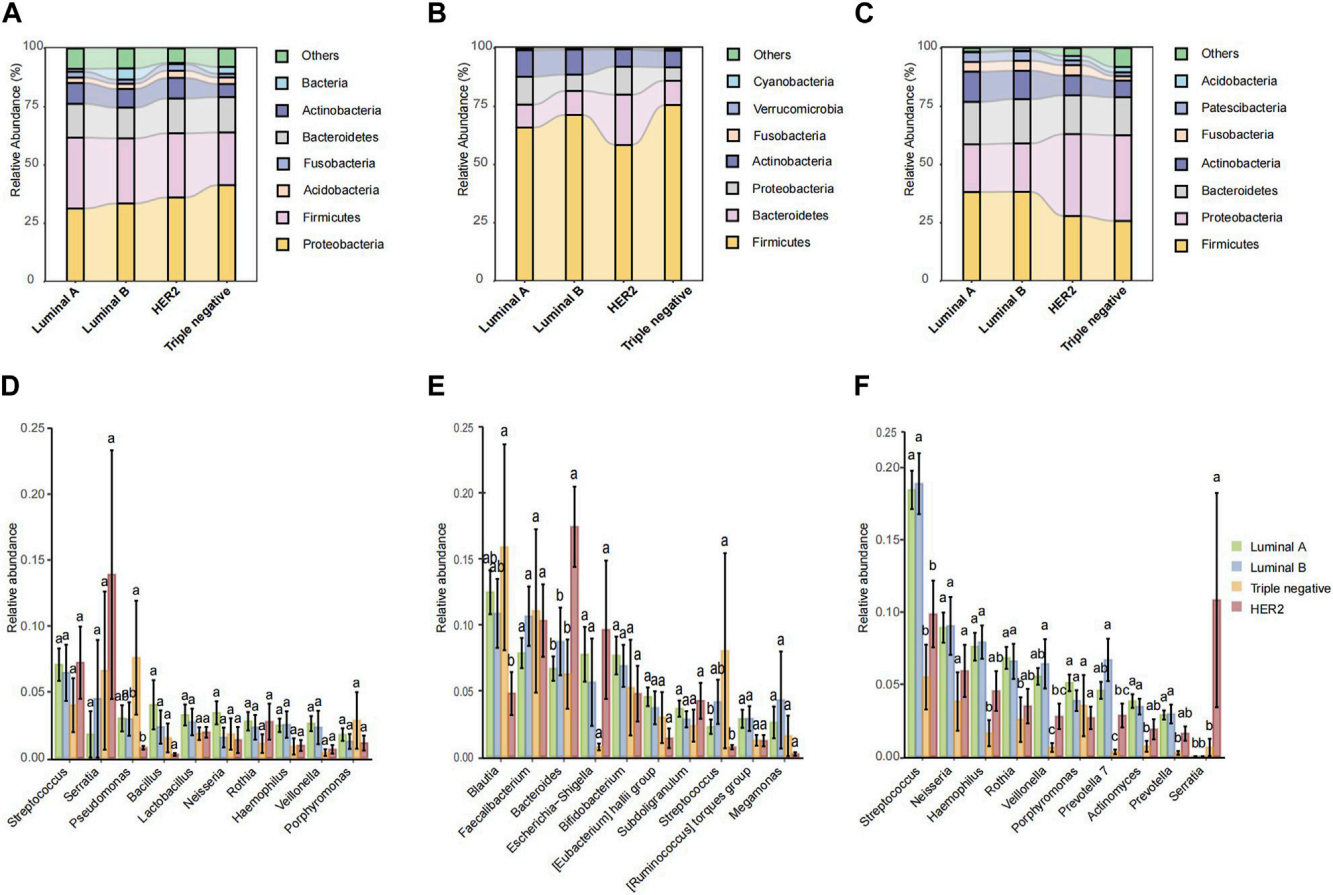
The microbiota relative abundances of all groups. **(A–C)** Stacked bar plot of mean proportions of breast, fecal and oral derived taxonomic composition of four types of breast cancer at genus levels. **(D–F)** Statistically differential genera of breast, fecal and saliva microbiota were evaluated with box plots. Different small letters in the bar chart represent statistical differences among the four groups.

Microbial species composition and relative abundance were investigated across various pathological types within breast tissue, fecal, and saliva samples. In breast tissue samples (Figure 3A), microbial structure similarity was observed between Luminal A and Luminal B, with Firmicutes and Bacteroidetes predominating. However, the HER2 type exhibited an increased abundance of Proteobacteria, while the TNBC type showed a slight increase in Actinobacteria. In fecal samples (Figure 3B), Luminal A displayed higher abundances of Verrucomicrobia and Cyanobacteria compared to other pathological types. Luminal B exhibited increased levels of Fusobacteria, while Triple negative showed a relative increase in Actinobacteria. In saliva samples (Figure 3C), both Luminal A and Luminal B showed a relative abundance of Bacteroidetes and Firmicutes, whereas HER2 and Triple negative types exhibited higher levels of Proteobacteria.

In the microbial abundance differential analysis, distinct variations were observed across the pathological types. Within breast tissue samples (Figure 3D), significant variations in *Streptococcus* and *Lactobacillus* abundance were noted across all pathological types, with HER2 and Triple negative types exhibiting higher levels of *Pseudomonas*. In fecal samples (Figure 3E), Luminal B exhibited a notably higher abundance of *Bacteroides* compared to Luminal A and HER2, while TNBC showed the lowest abundance of Faecalibacterium. Additionally, Luminal B and HER2 types showed an increased abundance of *Escherichia*. In saliva samples (Figure 3F), *Streptococcus* was highly abundant across all pathological types, though slightly less in TNBC; *Neisseria* was the most abundant in Luminal A and least in Triple negative. Conversely, HER2 type exhibited a higher abundance of Porphyromonas.

The Circos plot reveals the association strength between four pathological types of breast cancer and major microbes in three samples. The present study aimed to examine the relationships between microbial genera and breast cancer pathology kinds using three separate biological materials, namely, breast tissue, fecal, and saliva. Our findings consistently demonstrated both consistent and diverse patterns. The presence of Proteobacteria demonstrated a constant and significant relationship with the Luminal B subtype of breast cancer in all examined samples.

**FIGURE 4:**
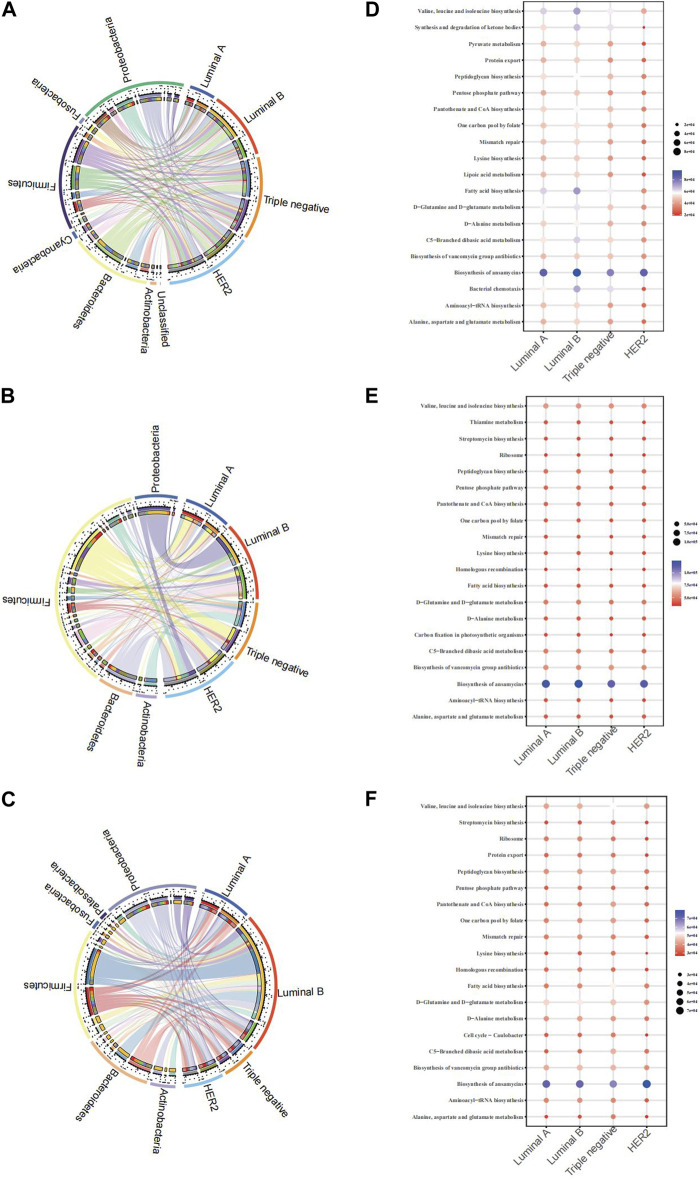
Chord diagram of the microbiota and BC pathological types of breast cancer and microbiome function prediction according to the KEGG pathway database **(A)** Chord diagram of breast tissue **(B)** Chord diagram of fecal samples **(C)** Chord diagram of saliva samples **(D)** KEGG for breast tissue **(E)** KEGG for fecal samples **(F)** saliva samples.

From the breast tissue samples (Figure 4A), there’s a pronounced affiliation between Luminal A and microbial communities such as Proteobacteria and Bacteroidetes. Notably, the TNBC subtype showcases a remarkable association with Actinobacteria, hinting at the potential role of specific microbes within breast tissue in relation to certain cancer subtypes. In the fecal samples (Figure 4B), we observe a differentiated microbial landscape. The bond between HER2 and Bacteroidetes emerges prominently, while Luminal B intertwines closely with Actinobacteria and Proteobacteria. This underscores the possibility that the fecal microbiota-breast cancer relationship possesses its own unique dynamics, distinct from that in breast tissue. Within the saliva samples (Figure 4C), Luminal B’s ties with both Proteobacteria and Bacteroidetes shine through, complemented by the evident relationship between Triple-negative and Actinobacteria. This alludes to the theory that saliva’s microbial matrix might offer specific insights into certain breast cancer subtypes. In analyzing KEGG functional prediction bubble charts across varied pathological breast cancer microbiomes, breast tissue samples chiefly exhibit lower abundance in most metabolic pathways, particularly within Luminal A and Luminal B types, most prominently in the pathways of “Biosynthesis of ansamycins” and “Alanine, aspartate, and glutamate metabolism” (Figure 4D). Fecal samples demonstrate a significant variation in the “Synthesis and degradation of ketone bodies” pathway, particularly associated with the HER2 type (Figure 4E). Saliva samples indicate a higher abundance of the “Biosynthesis of ansamycins” pathway in Triple-negative breast cancer (Figure 4F). Collectively, this data suggests potential variations in specific metabolic pathways within the microbiome across different breast cancer types, offering a potential avenue for further biological exploration and insights into breast cancer pathogenesis.

## 5 Discussion

The relationship between host microbiota and breast cancer is complex and multi-layered. Recent studies have greatly enhanced our understanding of dysbiosis as a factor contributing to breast cancer development. Studies show that unique shifts in the composition and function of the mammary and gut microbiomes in breast cancer patients may act as early biomarkers for tumor development (Alpuim Costa et al., 2021). Research indicates that tumor microbiota complexity varies according to breast cancer subtypes, stages, and racial and ethnic groups, suggesting a detailed interplay between microbial composition and genetic and environmental factors. The tumor microenvironment may harbor microbial populations, yet the relationship is bidirectional. Bacteria can promote tumor progression; conversely, tumor progression can lead to bacterial dysbiosis, making it challenging to discern the interactions’ causality and direction (Gilbert et al., 2018). Certain microbes are associated with genetic alterations in host cells that may influence cancer progression *Enterococcus faecalis* and *Staphylococcus* hominis exhibit anti-cancer properties by notably reducing cancer cell growth without harming normal cells (Hassan et al., 2016). *Lactobacillus* species can downregulate genes linked to aggressive breast tumor pathways, indicating a potential shift toward microbial-based cancer therapies (Riaz Rajoka et al., 2019). Conversely, microbes such as *Fusobacterium* nucleatum may promote tumor growth and metastasis, (Li et al., 2023; Van der Merwe et al., 2021) reflecting the microbiome’s dual role in breast cancer pathology. Urbaniak et al.’s groundbreaking research has revealed a unique breast tissue microbiome in cancer patients and shown that certain microbes can cause DNA damage, pointing to a direct microbial role in cancer development (Urbaniak et al., 2016). These findings emphasize the microbiome’s role in breast cancer development and propose microbiome modification as a treatment strategy. The potential for oral microbiota’s involvement in breast health is also being investigated, as seen in a Ghanaian case-control study. A case-control study conducted in Ghana explored the association between oral microbiota and breast cancer, as well as benign breast diseases, establishing a connection between the microbiota of the oral cavity and that of the fecal matter. This investigation highlights the potential systemic interplay between different microbial communities within the body and their collective impact on breast health (Wu et al., 2022). Key studies have identified microbial families associated with BC, emphasizing the role of microbiota in BC susceptibility and progression. German et al. (German et al., 2023) analyzed the microbiome of breast tissue from 403 women without cancer and 76 with breast cancer. They discovered a potential association between the presence of Lactobacillaceae, Acetobacterraceae, Xanthomonadaceae, and Ralstonia and breast cancer development. Additionally, analysis of transcriptome data from normal breast tissue revealed that a high abundance of *Acetobacter* aceti, *Lactobacillus* vini, *Lactobacillus* paracasei, and Xanthonomas sp. correlates with enriched metabolism and immune-related genes. Bacteroidetes, Firmicutes, Proteobacteria, and Actinobacteria were also identified by Klann et al. (Klann et al., 2020). Liu et al. (Liu et al., 2023) performed a 16S RNA analysis on 70 FFPE samples, identifying Bacteroidetes, Firmicutes, and Proteobacteria as the primary differential microbes. Additionally, in ER+/HER2-, ER+/HER2+, and ER-/HER2+ tumors, the genera Prevotella_9, *Bacteroides*, and Alloprevotella were the most active, whereas *Lactobacillus* showed heightened activity in triple-negative samples. Our study also validated the presence of differential microbes across these distinct pathological types of breast cancer.

The current investigation meticulously navigates through the symbiotic nexus between host microbiota, sprawling across diverse anatomical realms, and BC pathogenesis. By conducting an extensive appraisal of microbial compositions within fecal, saliva, and breast tissue specimens, spanning various BC pathological spectrums, the study elucidates the potential microbial underpinnings in BC onset and progression. Through a robust methodological framework, this research pioneers in unraveling the microbial intricacies, propelling a significant stride towards a profound understanding of BC’s microbial etiology.

The discernible variances in microbial diversity between individuals afflicted with BC and those with non-malignant breast conditions underscore the pivotal role of microbiota in BC pathogenesis. The significant disparities in alpha and beta diversity indices within breast tissue samples between the two cohorts accentuate the plausible influence of microbial consortia on BC susceptibility. Also, the analysis conducted using PICRUSt2 for KEGG pathway prediction indicated disparities in carbohydrate and inositol metabolism, consistent with the findings reported by German et al., with the presence of Ralstonia is linked to the dysregulation of genes in the carbohydrate metabolism pathway (German et al., 2023). Transitioning into the exploration of microbial compositions across various BC subtypes, a meticulous examination unveils a distinct microbial blueprint associated with each pathological subtype, hinting at the microbial predilections towards specific BC subtypes. For instance, a higher abundance of Proteobacteria in HER2 and Actinobacteria in Triple Negative cases reveal the intricate microbial-tumor crosstalk, which potentially modulates tumor behavior and thereby, the clinical outcomes. The review article by Rizzatti et al. emphasizes that Proteobacteria play a role not only in intestinal diseases but also may be related to extra-intestinal diseases, suggesting they might be a microbial signature of some diseases, including breast cancer (Rizzatti et al., 2017).

Expanding the scope to different anatomical niches, the study delineates the distinct microbial landscapes across the breast tissue, fecal, and saliva specimens. This underlines the potential of employing a multi-niche microbial analysis approach in deciphering the complex microbial dynamics in BC pathogenesis. For instance, the pronounced affiliation between the Luminal A subtype and Proteobacteria and Bacteroidetes within breast tissue, contrasted by different microbial associations within fecal and saliva samples, elucidates the necessity of a multi-dimensional microbial analysis for a nuanced understanding of BC-microbiota interactions.

This study, exploring the link between microbial communities and breast cancer, holds certain limitations. The small sample size and single-center approach may hinder generalizability, necessitating larger, multi-center studies in the future. The focus on specific microbial taxa might not provide a comprehensive microbial landscape, calling for deeper microbial analysis using advanced technologies. The absence of long-term follow-up data leaves the impact of microbial variations on clinical outcomes unexplored. Unaccounted potential confounders like dietary habits could influence the findings. The study also does not explore microbial intervention effects on treatment efficacy, nor delves into the molecular mechanisms involved, suggesting avenues for future in-depth investigations.

Looking forward, the findings furnish a vital foundation for future explorations aimed at elucidating the precise microbial interactions in BC ontogenesis. The potential of microbial profiling in early BC detection and monitoring, along with its prognostic and therapeutic implications, merits further in-depth investigations. Moreover, the substantial microbial divergences across different BC subtypes advocate for a more personalized microbial analysis approach in BC management. Future studies should pivot to extensive longitudinal research, engaging larger and more varied populations, and harnessing omics and translational methodologies to fully elucidate the microbiome’s role in breast cancer (BC). This research has the potential to shape innovative predictive and preventive strategies, as well as personalized treatment protocols that may include microbial modulation. The dynamic relationship between microbial communities and the immune system, alongside the capacity of certain microbes to either induce genetic instability or offer therapeutic advantages, presents promising avenues for investigation. Embracing a precision medicine framework could transform BC management by integrating microbial, genomic, and environmental data to customize prevention and treatment for each patient’s unique profile.

## 6 Conclusion

The study highlights the intricate connection between microbial structures, their functional roles, and the diversity observed among different subtypes of breast cancer. Further investigation is required to fully comprehend the possible contributions of these microbial organisms to the dynamics of breast cancer.

## Data Availability

The raw data supporting the conclusion of this article will be made available by the authors, without undue reservation.
