# Supplementary material for: Relationships among breast, gut, and oral microbiota across diverse pathological types of breast cancer, a Chinese cohort study
Source: Front Mol Biosci. 2023 Nov 21;10:1325552. doi: 10.3389/fmolb.2023.1325552 (PMC10699584; doi:10.3389/fmolb.2023.1325552)
Supplement: Supplementary file 1 [file Table1.DOCX]

Supplementary Material

**Relationships Among Breast, Gut, and Oral Microbiota Across Diverse Pathological Types of Breast Cancer, a Chinese Cohort Study**

**Kexin Feng^1^, Fei Ren^1^, Xiang Wang^1*^**

^1^Department of Breast Surgical Oncology, National Cancer Center/National Clinical Research Center for Cancer/Cancer Hospital, Chinese Academy of Medical Sciences and Peking Union Medical College, Beijing, 100021, China

*** Correspondence:**

Xiang Wang, xiangw630505@163.com

# Supplementary Figures

## Supplementary Figures


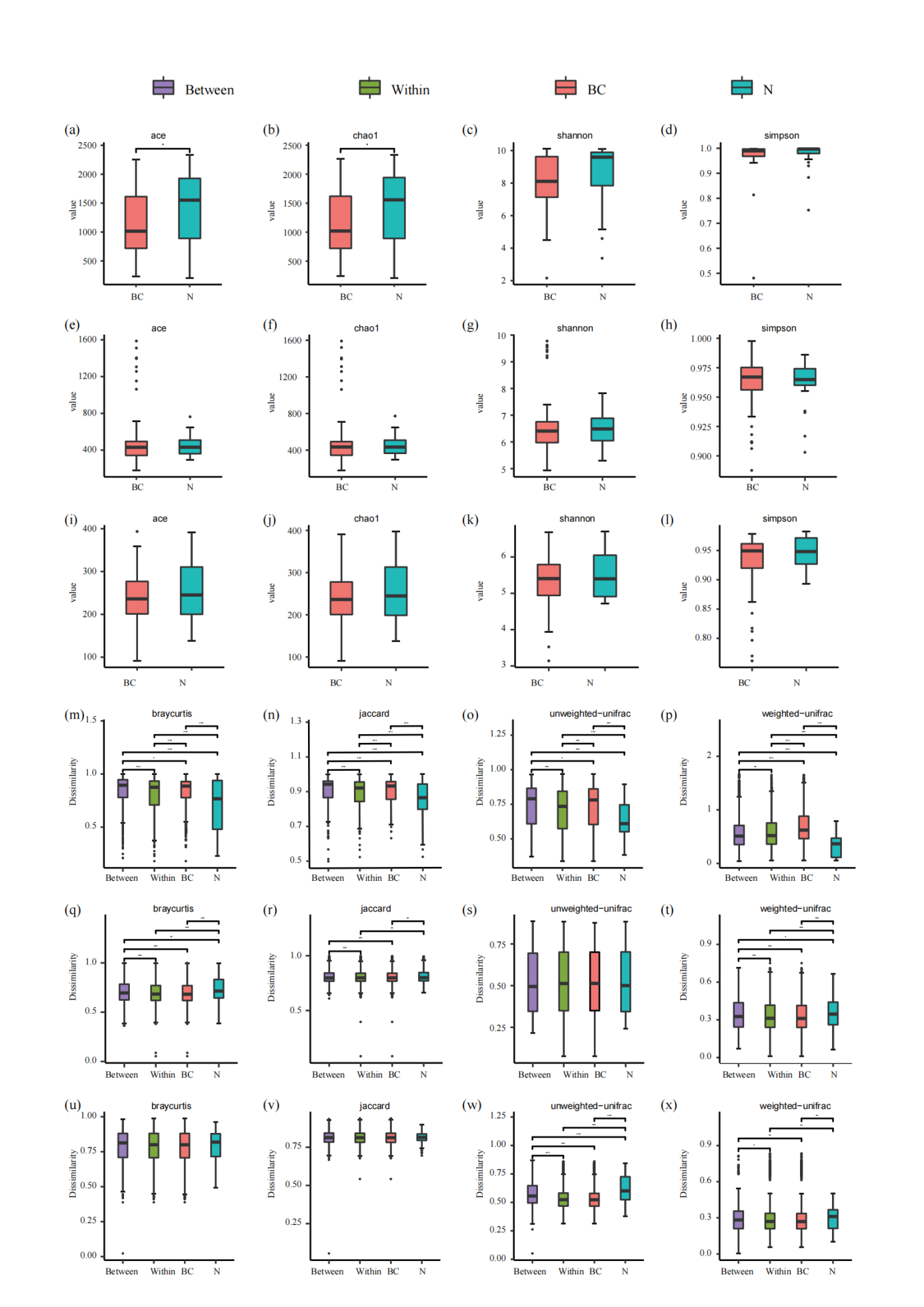


**Supplementary Figure 1.** Indexes of alpha diversity(α-diversity) and beta diversity (β-diversity) of the microbiota in breast tissues, fecal samples and saliva samples (a-d, α-diversity of breast tissues; e-h, α-diversity of fecal samples; i-l, α-diversity of saliva samples; m-p, β-diversity of breast tissues; q-t, β-diversity of fecal samples; u-x, β-diversity of saliva samples)


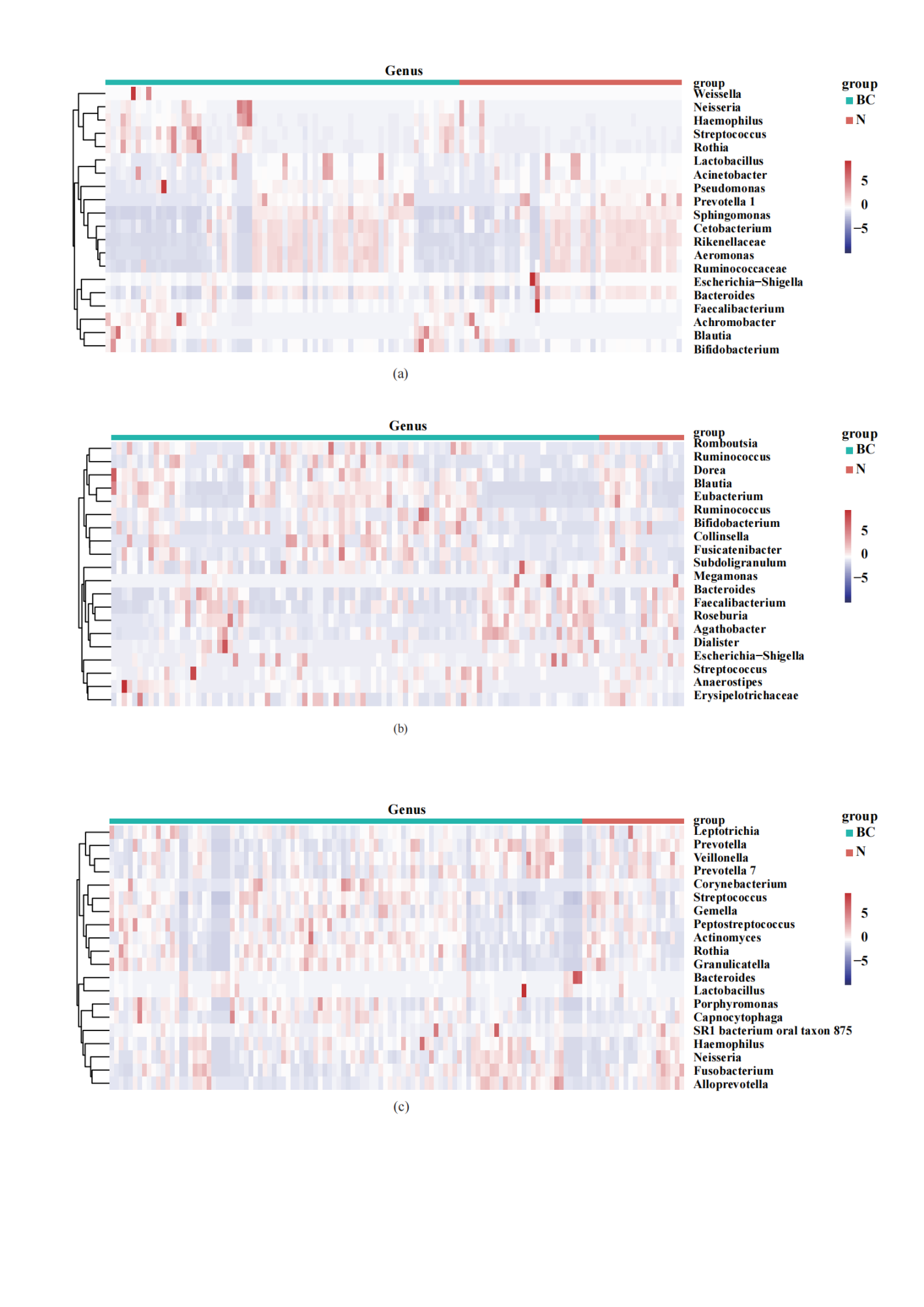


**Supplementary Figure 2.** Heatmaps at the genus level of the microbiota in breast tissues, fecal samples and saliva samples.(a) breast tissues; (b) fecal samples; (c) saliva samples
